# Supplementary material for: Habitat remediation followed by managed connectivity reduces unwanted changes in evolutionary trajectory of high extirpation risk populations
Source: PLoS One. 2024 May 30;19(5):e0304276. doi: 10.1371/journal.pone.0304276 (PMC11139274; doi:10.1371/journal.pone.0304276)
Supplement: S1 Methods — Here, we provide additional information about each model function. (DOCX) [file pone.0304276.s001.docx]

**S1 Methods**

We created an agent-based model to predict long term evolutionary trends by introducing various migration rates in populations with varying levels of extinction risk. Below, we provide additional information about each model function.

Initialize recipient and source populations
Simulations began with the recipient population at carrying capacity (K = 1000) and a large source population (K_S_ = 5000). Individuals in both populations were initialized in a similar way; individuals were assigned an individualized ID number, age according to a Poisson distribution (rpois function in R) minus one, and a random sex (0 = female, 1 = male). Additionally, all individuals were characterized by three sets of SNPs: (1) neutral and assigned with varying minor allele frequencies (nSNP_D_ = 1000; 0.05 ≤ p ≤ 0.15 or 0.40 ≤ p ≤ 0.50), (2) fixed for different alleles in each population (nSNP_M_ = 100) so that the recipient population was homozygous for one allele (00) and the source population was homozygous for the alternative allele (11), and (3) fixed for the same allele (00) in both populations (nSNP_C_ = 100). Therefore, a total of 2400 alleles describe each individual, with each locus defined as heterozygous (01) or homozygous (either 00 or 11) and designed to measure specific population characteristics. Relative fitness for all individuals was calculated as the observed heterozygosity across the nSNP_D_ loci. Similarly, the proportion of alleles from migrant ancestry was calculated across the nSNP_M_ loci for all individuals. Mutations across nSNP_C_ loci were tracked and considered a deleterious recessive mutation if both alleles at a single locus mutated. When the mutate_init function was active, mutations in the initialized recipient and source populations varied across SNP types (µ = 2.2 x 10^-9^ in nSNP_D_; µ = 4.8 x 10^-10^ in nSNP_C_). Further, when the mutate function was active, mutations in offspring occurred with the same probability of the initialized population in all SNPs. When mutation occurred, alleles passed from parent to offspring were switched to the alternative allele state. The recipient population then went through each of the life history events described below yearly for a total of 350 years.

Age Up
The first step in the model involved incrementing the age of all surviving individuals by one year. Because this is the first step in the model, the initialized population consisted of individuals that would be “born” in the first year.

Death
There were three functions that removed individuals from the population. Following the aging of individuals, a fitness-induced chance of death was imposed on the individual at the age of maturity equal to the inverse of the number of deleterious mutations in nSNP_C_ within the individual so that individuals with a greater number of deleterious mutations (i.e., both alleles mutated from 00 to 11 at the same locus) had a lower fitness and an increased chance of mortality. Additionally, as the last step in each simulation year, we assumed the cumulative probability of death of an individual was equal to the quotient of an individual’s age and the maximum lifespan. Finally, in each year, individuals were faced with a chance of mortality equal to the inverse of the total number of mutations in nSNP_C_ so that increased mutations increased the probability of death. It is important to note that mutations only occurred at birth of the individual such that there is an equal probability of death due to mutation at all ages of the individual’s lifespan. Individuals that were forced into mortality were removed from subsequent model steps.

Migrate
Migrants were randomly selected from the source population to move into the recipient population, with the number and timing of migrants per generation selected determined based on the parameter set. Although migrants move into the recipient population and were preferentially chosen for mating pairs (when the matemigs function was active), the number of migrants in the population are not exactly equal to the number of effective migrants in that generation since the number of migrant pairs could be more than the number of offspring generated in that generation or, at small population sizes, opposite sex individuals may not be available.

Mate Choice
Reproduction occurred between randomly selected pairs of adults, with preference given to migrants (when matemigs was active). Parents of the opposite sex were matched as mates with replacement so that individuals could mate with more than one individual in that year. An Allee effect was imposed (when allee was active) so that as the number of individuals in the population decreased, the chance of mates interacting decreased; the probability of finding a mate was the complement of the reciprocal of the total number of adult pairs.

Setting Population Size
The number of offspring produced per year was determined using the logistic growth equation so that the total number of individuals in the next year was calculated. The recipient population was allowed to persist around carrying capacity for the first 100 years. Following that 100-year period, the population was forced through a bottleneck for 10 years, persisted at a lower carrying capacity for 40 years, and then allowed to expand at a rate of logistic growth up to the original carrying capacity of 1000 individuals. Logistic growth was calculated with the equation

$N_{t+1}=\left( 1+r\left( 1-\frac{N_{t}}{K} \right) \right)* N_{t}$ (1)

where the population size $(N_{t+1})$ was determined by the per capita growth rate (*r* = 1), carrying capacity (K = 1000), and the population size prior to reproduction (*N_t_*). The size of the carrying capacity at the duration of the bottleneck (K_B_) was quantified so that the new carrying capacity would be within limits for IUCN criteria for the evaluation for the Red List. Specifically, IUCN criteria evaluate species that are vulnerable as those that have > 10% decline in 10 years (K_B_ = 700), endangered with a 50-70% decline in 10 years (K_B_ = 300), and critically endangered with a 80-90% decline in 10 years (K_B_ = 100).

Reproduction
The number of offspring needed to maintain the population size generated in the PopSizeNext function was used to determine how many mates were required to create enough offspring with a maximum fecundity between parental pairs (broodsize = 2). If matemigs was active, parental pairs with at least one migrant were preferentially chosen to generate offspring while additional pairs were randomly selected. Offspring genotypes were assigned according to Mendelian inheritance, where one allele at each locus was randomly selected from each parent. If mutate was active, mutation on generated genotypes occurred at a rate of 2.2 x 10^-9^ mutant/generation/allele in nSNP_D_ and 4.8 x 10^-10^ mutant/generation/allele in nSNP_C_. If an allele was selected to mutate, the allele was switched from either a 0 to a 1 or a 1 to a 0, depending on the initial identity.

Analyze
After each generation, we calculated population demographics to monitor the population. Demographic calculations included the number of effective migrants, the number of effective parents, total number of individuals, the sex ratio, and the number of adults. The number of migrants in the population and the proportion of migrant SNPs (nSNP_M_) allowed us to monitor how migrant alleles were distributed across individuals. Genetic diversity and fitness were classified as the observed heterozygosity, calculated across all SNPs (nSNP_D_ + nSNP_M_ + nSNP_C_) and in drift SNPs (nSNP_D_). Inbreeding in the population (F_IS_) and the amount of divergence in the recipient population (F_ST_) at that year as compared to the source population and compared to the initialized recipient population were evaluated using the *hierfstat* package in R (version 0.5-11). After all simulation years, all dead individuals were written back into the population to calculate the lifetime (LRS) of all individuals by evaluating the number of mates, number of offspring, and the number of offspring that survived to maturity for all individuals.

Lifetime Reproductive Success
Prior to migration of individuals into the recipient population, we assumed there was no prior history of reproductive success for migrants. Therefore, at the conclusion of each replicate of the simulation, lifetime reproductive success was calculated by determining the sum of offspring that were generated and survived to maturity by each parent while living in the recipient population.

Quantitative Comparisons
We compared the effect of all parameters on genetic diversity and population persistence by comparing the output of the simulated populations to control simulations. We considered outputs to be similar when confidence intervals (CIs) overlapped and significantly different when there were no overlapping CIs. For each parameter set, we measured the heterozygosity and the distribution of each SNP type to examine how migration altered the genetic diversity of the recipient population. Additionally, we evaluated pairwise F_ST_ values each year compared to the initialized recipient and source populations. Population size, inbreeding level (F_IS_), and lifetime reproductive success were also calculated. We ran 100 replicates for each combination of parameters, with each simulation run in R (version 4.2.1) on a high-performance computing cluster and visualized results in RStudio (version 2023.12.1+402) using the *scales* package (version 1.2.1).
