# Supplementary figures and images for: Habitat remediation followed by managed connectivity reduces unwanted changes in evolutionary trajectory of high extirpation risk populations
Source: PLoS One. 2024 May 30;19(5):e0304276. doi: 10.1371/journal.pone.0304276 (PMC11139274; doi:10.1371/journal.pone.0304276)

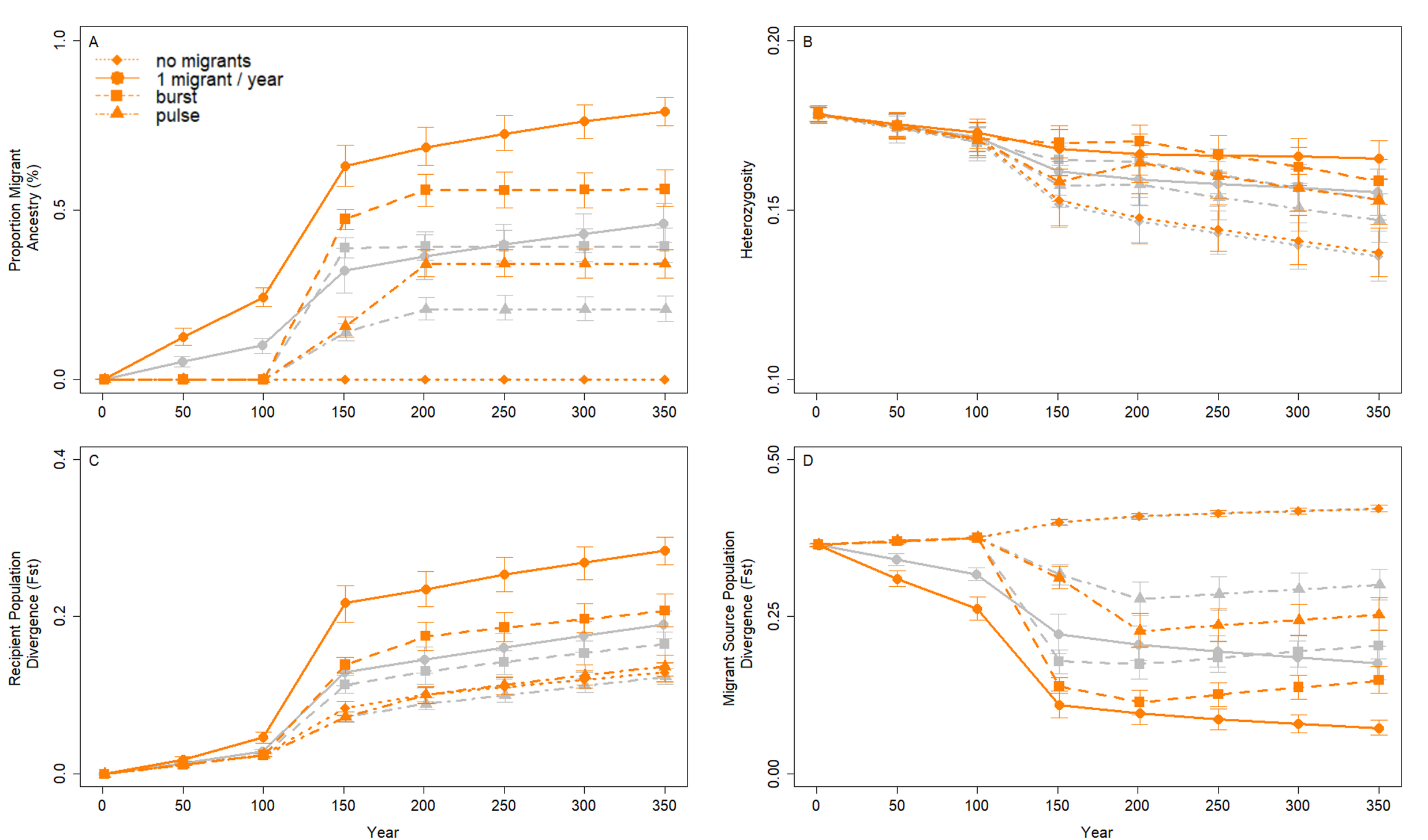

Supplement: S1 Fig — Movement of a single individual per year (solid line), burst migration of 100 individuals once (year 151; dashed line), and four pulse migrations of 25 individuals (years 151, 165, 181, 195; dashed and dotted line) was used to support and bolster these populations through remediation (year 151) and population recovery. These trends were compared to the same demographic patterns but without migration (dotted line). The proportion of migrant ancestry present in the recipient populations (A), observed heterozygosity (B), divergence of the recipient populations from the historical populations over time (C), and divergence of the recipient populations from the migrant source populations each year (D) illustrate the new evolutionary trends resulting from these migration decisions. Lines represent mean values across 100 replicates and error bars represent the confidence intervals needed to compare among parameter sets assuming alpha = 0.05 (i.e., 95% confidence intervals). (TIF) [file pone.0304276.s002.tif]

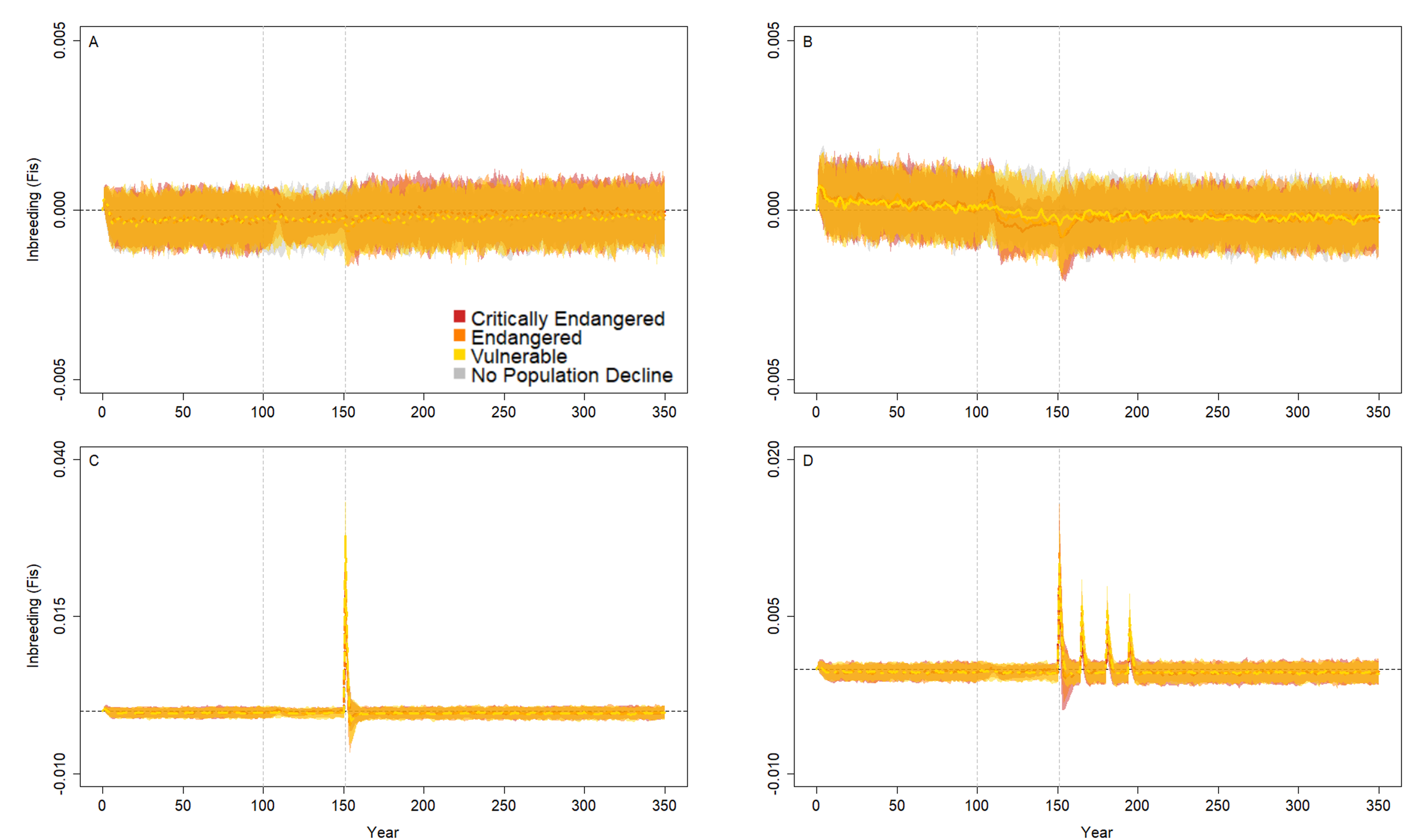

Supplement: S2 Fig — Note that the y-axis differs within this figure. Grey vertical lines depict the years at the start of population decline (y = 100) and subsequent habitat restoration (y = 150). The black horizontal line shows when FIS is zero. Lines represent mean values across 100 replicates and shaded areas represent the confidence intervals needed to compare among parameter sets assuming alpha = 0.05 (i.e., 95% confidence intervals). (TIF) [file pone.0304276.s003.tif]

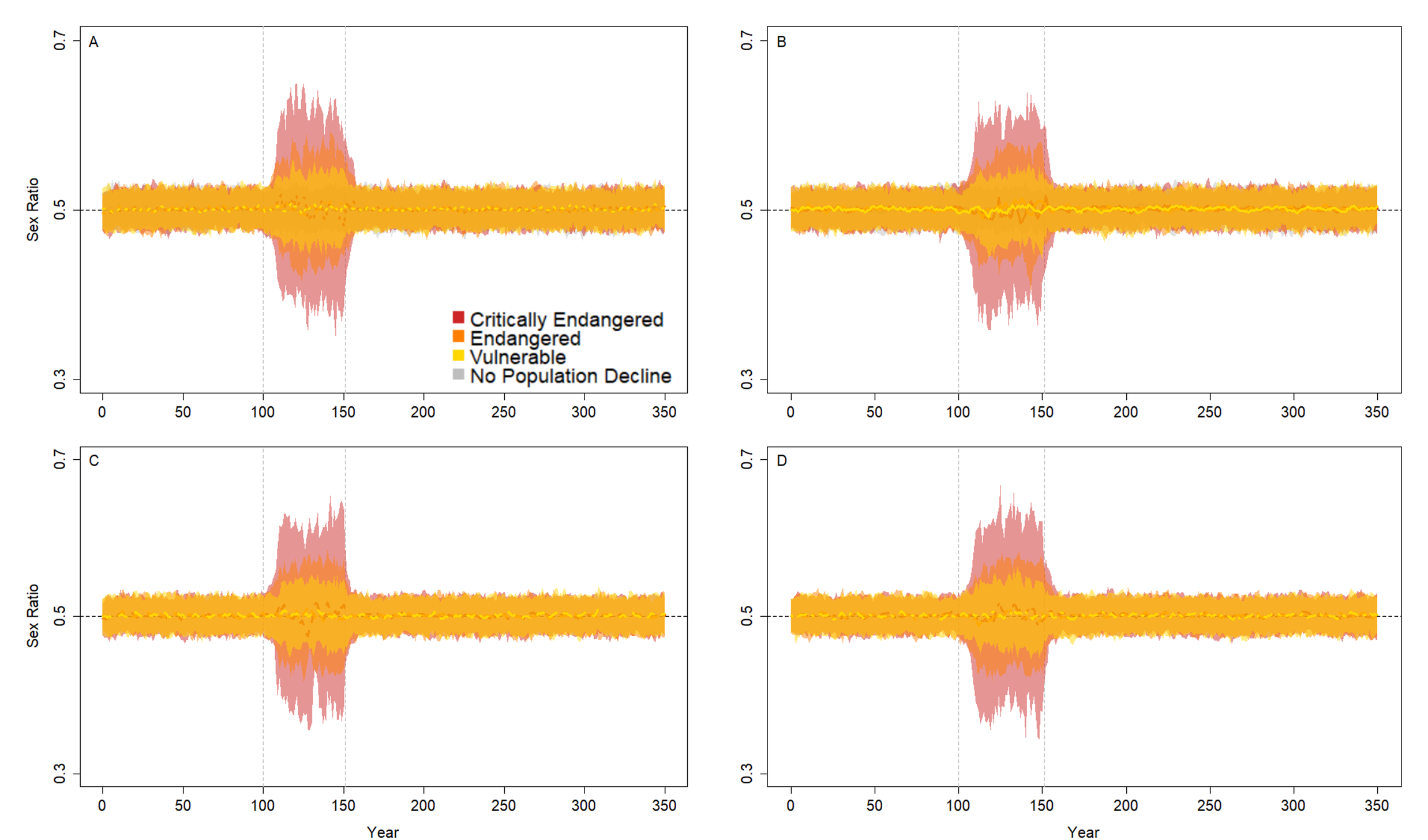

Supplement: S3 Fig — Grey vertical lines depict the years at the start of population decline (y = 100) and subsequent habitat restoration (y = 150). The black horizontal line depicts a 50:50 sex ratio; the population is female dominated when the ratio < 0.5 and male dominated when the ratio > 0.5. Lines represent mean values across 100 replicates and shaded areas represent the confidence intervals needed to compare among parameter sets assuming alpha = 0.05 (i.e., 95% confidence intervals). (TIF) [file pone.0304276.s004.tif]

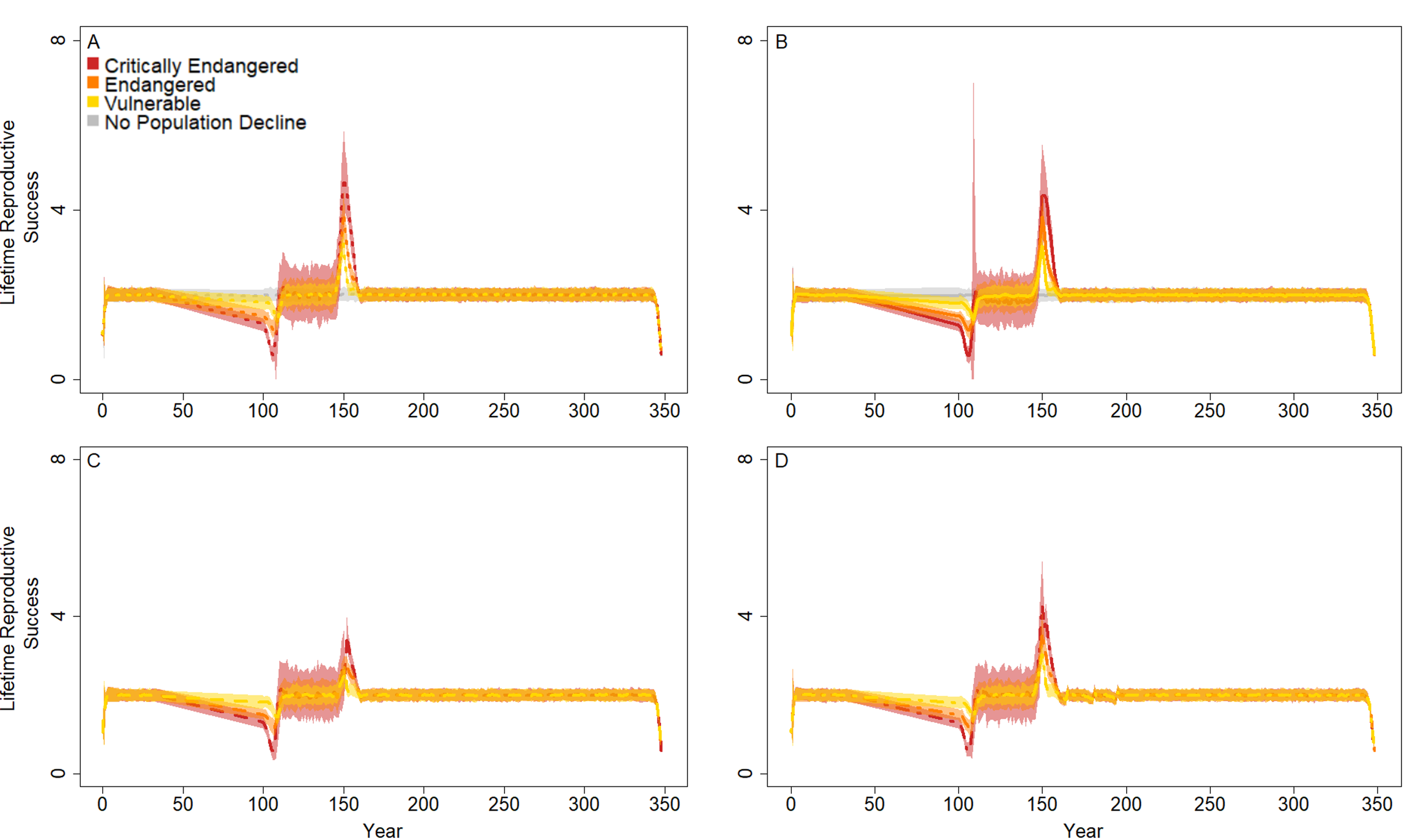

Supplement: S4 Fig — Lines represent mean values across 100 replicates and shaded areas represent the confidence intervals needed to compare among parameter sets assuming alpha = 0.05 (i.e., 95% confidence intervals). (TIF) [file pone.0304276.s005.tif]
